# Grafting as a strategy to increase flowering of cassava

**DOI:** 10.1016/j.scienta.2018.06.070

**Published:** 2018-10-20

**Authors:** Leonardo Silva Souza, Rafael Parreira Diniz, Reizaluamar de Jesus Neves, Alfredo Augusto Cunha Alves, Eder Jorge de Oliveira

**Affiliations:** a Universidade Federal do Recôncavo da Bahia, Campus Cruz das Almas, 44380-000 Cruz das Almas, BA, Brazil; b Embrapa Mandioca e Fruticultura, Rua da Embrapa, Caixa Postal 007, 44380-000 Cruz das Almas, BA, Brazil

**Keywords:** Propagation, Seed set, *Manihot* spp, Flowering induction

## Abstract

In cassava (*Manihot esculenta* Crantz), transferring genes via genetic breeding depends on crosses between contrasting progenitors, which is often limited by the low flowering rate of many genotypes. The main purpose of this work was to evaluate the effect of grafting on floral induction of cassava. For this, three genotypes were used: 1) BRS Formosa: a genotype with low flowering rate; 2) BGM0823: a genotype with high flowering rate; and 3) FLA05-02: a genotype of *M. esculenta* ssp. *flabellifolia* with high flowering rate. Cleft grafting was performed to generate the following treatments: Self-grafting of: 1) BGM0823 (Self-0823); 2) BRS Formosa (Self-Formosa); and 3) FLA05-02 (Self-FLA); and grafting of the genotypes, with the first being the scion and the second the rootstock: 4) BGM0823 × BRS Formosa; 5) BGM0823 × FLA05-02; 6) FLA05-02 × BRS Formosa; 7) FLA05-02 × BGM0823; 8) BRS Formosa × BGM0823; 9) BRS Formosa × FLA05-02; and also ungrafted treatments: 10) BGM0823; 11) BRS Formosa; and 12) FLA05-02. The results showed a 201% increase in the production of male flowers, 560% of female flowers and 400% of fruits in BRS Formosa grafted on BGM0823. BGM0823 (rootstock) also increased fruit production by 190% of FLA05-02. The grafted cassava plants exhibited an increase on the shoot production, although there was no change in the fresh root yield. The grafting of genotypes with high flowering rates can induce flowering in genotypes with low flowering rates.

## Highlights

• Grafting can transfer floral stimulus between different cassava genotypes.
• Grafting in early stage increase plant survival and favor the flowering induction.
• High fruit set were found in low flowering genotypes when grafted onto high flowering ones.
• The use of grafting in cassava affected the aboveground yield and harvest index.

## 1 Introduction

Cassava (*Manihot esculenta* Crantz) has gained importance in recent decades due to its many forms of use: as human food, in which it is considered a food security crop and is part of the diet of 800 million people in the tropics (El-Sharkawy, 2012); as animal feed; and for various industrial uses, including to make biofuels. Therefore, cassava breeding programs in Brazil and other countries in Latin America and Africa have increased their research with the objective of obtaining superior genotypes that meet the specific demands of farmers and industries.

For the selection of high performance genotypes, it is necessary to increase the genetic variability, which can be done through hybridization or self-fertilization in order to produce seeds. However, among the major drawbacks of this process is the absence or reduced flowering rate observed in most genotypes and the lack of synchronization of flowering (Ceballos et al., 2017). The production of fertile flowers is a basic premise for plant breeding, but obtaining flowers in a synchronized manner and within a suitable time interval is a major challenge (McGarry et al., 2017), considering that some genotypes do not produce flowers (Ceballos et al., 2017).

Erect and late-branching plants are preferred by farmers due to the greater ease of crop handling and possibility of using mechanized planting systems. In general, these plants do not bloom or bloom poorly during the normal growing cycle. Thus, efforts to develop new cassava genotypes to meet the demands, especially for mechanized planting, tend to select plants that flower late and little. In other words, the use of erect or late-branching genotypes as progenitors in recurrent selection cycles results in limited production of seed, so it is necessary to search for alternatives to induce flowering.

Some physiological aspects help explain the difficulties of flowering of some genotypes. The transition from the vegetative to the reproductive stage depends on both endogenous (hormones) and environmental (temperature and photoperiod) signaling, determined by the differentiation of the apical meristem, where the cells undergo changes in development (Bernier and Périlleux, 2005). This change of the apical meristem can be induced by the exogenous application of growth regulators such as auxins, gibberellins, abscisic acid and ethylene (Yang et al., 2016). In addition, flowering is initiated from the floral signs, also called floral or florigen stimulus. Amasino (2010) studied these floral signals at the molecular level and found that the overexpression of the FT (florigen) gene accelerates the flowering rate in *Arabidopsis thaliana* plants. In turn, Adeyemo et al. (2011) characterized the EFL4 gene (MeELF4) and identified the circadian genes of the cassava photoperiod, because the plants responded to diurnal rhythms or photoperiodic changes and the MeELF4 gene exhibited its expression at dusk in several parts of the plant.

The floral signals are produced in response to day length and translocated from the leaves to the apical meristem, where the flower bud production is established (Corbesier and Coupland, 2005). The hypothesis that florigen can move from leaf to meristem was demonstrated in *Arabidopsis thaliana* and rice (Corbesier et al., 2007; Tamaki et al., 2007). In these studies, the results showed that most if not all changes associated with the transition from vegetative to reproductive stage induced by day length could be ascribed to florigen.

Considering that florigen moves from the leaf to the meristem, Ceballos et al. (2017) evaluated floral induction in cassava by grafting. Initially, grafting was performed using late blooming or non-flowering genotypes as scions and genotypes with high flowering rate as rootstocks, and it was found that none of the treatments produced branches or flowers. In a second step, after the development of the scions, their cuttings were planted in the field, where three phenotypic responses were observed: some genotypes did not branch and did not flower; other genotypes branched but produced no flowers; and finally, in one genotype there was branching with abundant production of flowers, fruits and seeds in relation to ungrafted stem plants. Therefore, the grafting technique is a viable tool for floral induction, since it can promote the transfer of mobile elements throughout the plant, such as water, nutrients, metabolites and proteins (Mudge et al., 2009). The knowledge that the initiation of floral development starts with the movement of florigen (signal), which is produced in the leaves and transported through the phloem to the apical meristem where the interaction with other factors occurs (Amasino, 2010; Ceballos et al., 2017), is an advance in the analysis of floral induction in cassava. In this sense, the use of abundant flowering rootstocks could transfer the flowering stimulus to scions that show low flowering rate.

In addition to grafting, other floral induction techniques have been mentioned in the literature, such as the insertion of a flowering promoter sequence by inoculation of the zucchini yellow mosaic virus in melon (*Cucumis melo* L.) (Lin et al., 2007) and floral induction in cotton after inoculation of the cotton leaf crumple virus (McGarry and Ayre, 2012). In cassava, new techniques have demonstrated the over-expression of *Arabidopsis* FLOWERING LOCUS T in *Agrobacterium*-mediated transformed cassava, triggering early flowering in glasshouse-grown plants (Bull et al., 2017). These authors reported success in the expression of the inserted sequence, so that the transgenic plants emitted flowers and produced fruits from four months after planting. Although floral induction in cassava can be done using different techniques, few institutions have the necessary mastery of gene manipulation to create, edit, and insert promoter sequences into the cassava genome. In addition, the use of transgenic cassava, especially in Brazil, is not yet regulated, so it will undergo a long process of standardization and biosafety studies, especially considering that the species is native to Brazil, where simple and efficient techniques to induce flowering should still be considered. Therefore, the objective of this work was to analyze the possibility of flower induction in cassava plants through flowering signal transmission using different scion-rootstock combinations of genotypes with low or high flowering rate.

## 2 Materials and methods

The experiments were carried out in two stages (nursery and field) at the experimental area of Embrapa Mandioca e Fruticultura (Embrapa Cassava & Fruits research unit) in Cruz das Almas, Bahia, located at coordinates 12°40′19″S and 39°06′22″W, 220 m above sea level, from February 2015 to April 2016.

### 2.1 Plant material

Three genotypes belonging to the Cassava Germplasm Bank (CGB) of Embrapa Mandioca e Fruticultura were selected: 1) BRS Formosa (*M. esculenta*), an elite genotype introduced by Embrapa that has a low flowering rate; 2) BGM0823 (*M. esculenta*), a genotype of the CGB that has a high natural flowering rate; and 3) FLA05-02, a genotype of *M. esculenta* ssp. *flabellifolia*, a subspecies that has a high flowering rate. *M. esculenta* ssp. *flabellifolia* was also selected due to the potential of interspecific crosses with *M. esculenta*, which is currently attracting the interest of breeders, especially for the introduction of resistance to pests and diseases.

In February 2015, about 200 stem cuttings (10–12 cm length) of BRS Formosa and BGM0823 were planted in polyethylene bags (10 cm × 25 cm, diameter × height), filled with potting mix containing soil, vermiculite and chicken manure (2:2:1, v:v). At the same time, about 400 open-pollinated seeds of the genotype FLA05-02 obtained in the CGB were planted in bags similar to those used for the cuttings. The planted materials were placed in a greenhouse with 50% shade, where the temperature and relative humidity varied from 20 to 35 C and 50 to 80%, respectively, during the period of seedling production.

### 2.2 Grafting

**Fig. 1:**
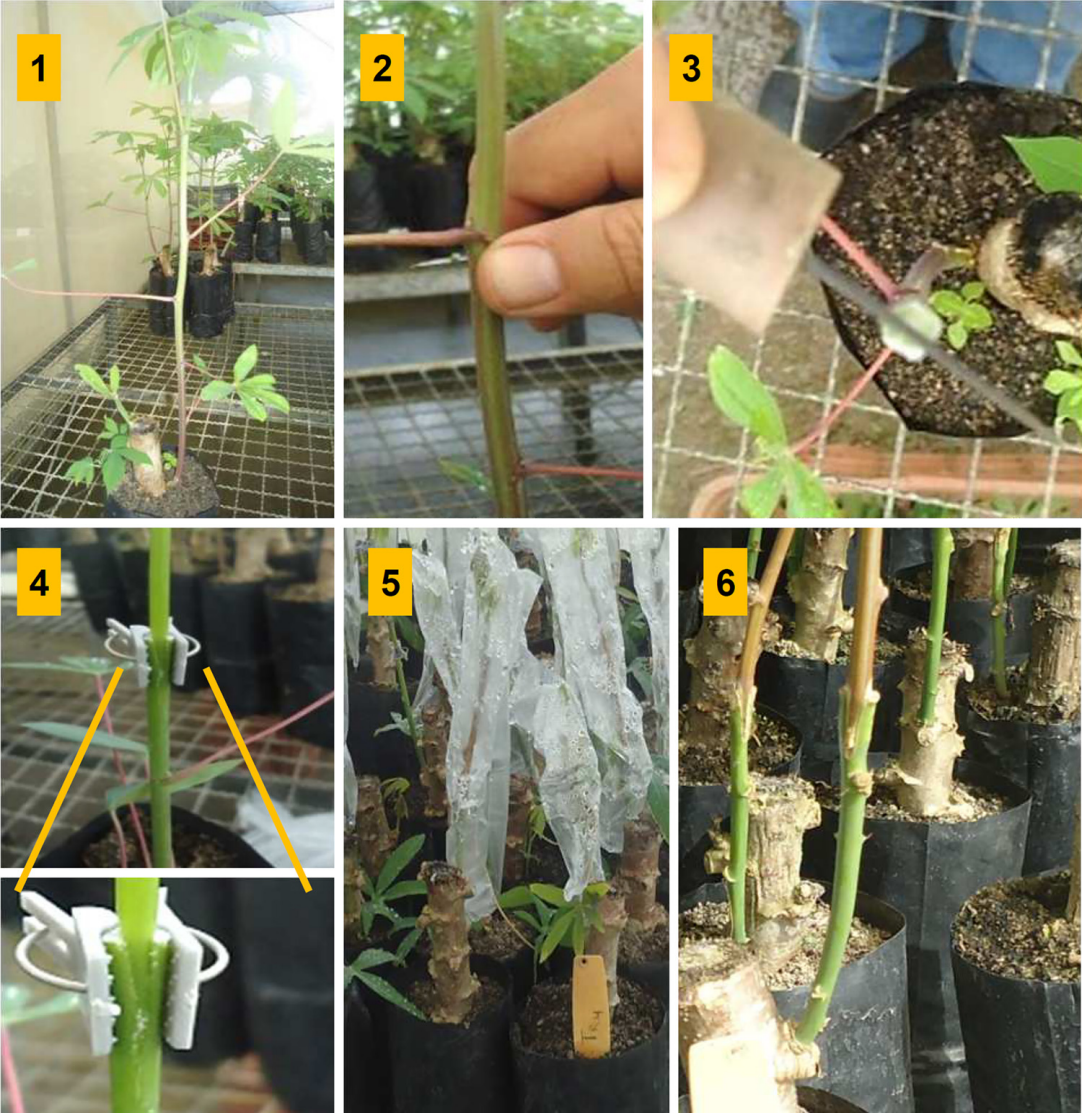
Cleft grafting procedure used: 1 = ungrafted plant with approximately 6 mm stem diameter; 2 = cut in the middle portion of the stem of the rootstock, leaving a pair of leaves at a height of 12 cm; 3 = opening of the longitudinal slot (1.0 cm) in the rootstock; 4 = stem grafted and fixed with a plastic clamp; 5 = pair covered with a transparent plastic bag; 6 = grafted plant.

The choice of grafting method was based on a pilot test carried out previously. In this test, three types of grafting were used: cleft grafting; splice grafting and normal “T” type budding. The graft survival rate was evaluated considering the production of seedlings with apparent adherence and good vigor. The cleft grafting presented higher survival rate and was used to perform grafts with different scion-rootstock combinations of the three selected genotypes. In summary, when the plants’ stems reached a diameter of approximately 6 mm, the seedlings were cut at medium height, leaving a pair of leaves in the rootstock at a height of approximately 12 cm (Fig. 1).

Then, we selected a stem with diameter similar to the rootstock for fork formation (grafting), and a longitudinal slot was opened (1.0 cm) in the rootstock. The scion, cut into a "V" shaped wedge, was inserted into the rootstock carefully to align with the cambial zone and secured with a small plastic clamp, and bagged to maintain high moisture (Fig. 1). Two leaves of the rootstock were maintained until the development of new leaves by the grafted part, which occurred approximately 15 days after grafting, when they were manually removed.

The rootstocks and scions produced by the vegetative propagation of the BRS Formosa and BGM0823 genotypes reached the grafting stage at 25 days after sprouting, while the rootstocks and scions of seminal origin (FLA05-02) reached the time for grafting 30 days after sowing. At the end of the grafting process, 12 treatments were obtained for the flowering analyses: 1) self-grafting of BGM0823 (Self-0823); 2) self-grafting of BRS Formosa (Self-Formosa); 3) self-grafting of FLA05-02 (Self-FLA); 4) BGM0823 as scion × BRS Formosa as rootstock (0823/Formosa); 5) BGM0823 as scion × FLA05-02 as rootstock (0823/FLA); 6) FLA05-02 as scion × BRS Formosa as rootstock (FLA/Formosa); 7) FLA05-02 as scion × BGM0823 as rootstock (FLA/0823); 8) BRS Formosa as scion × BGM0823 as rootstock (Formosa/0823); 9) BRS Formosa as scion × FLA05-02 as rootstock (Formosa/FLA); 10) BGM0823 ungrafted BGM0823 (0823); 11) BRS Formosa ungrafted (Formosa); and 12) FLA05-02 ungrafted FLA05-02 (FLA).

The plastic bag and clamp used to protect the grafting union were removed seven and twelve days after the grafting procedure, respectively. After grafting, the treatments were kept in a greenhouse, irrigated via micro sprinkler, for about 80 days.

### 2.3 Evaluation of the development of plants in the nursery

To verify the viability of the grafting, an experiment was performed with all treatments. The treatments were set up in a completely randomized design, in a simple factorial scheme of 3 (scions) × 3 (rootstocks), making nine combinations plus 3 ungrafted plants (12 treatments), with three replications of 20 plants.

At 30 days after the grafting procedure, we evaluated the survival rate considering the production of seedlings with apparent adherence, new leaves and good vigor. The data were transformed to percentage of survival (%). The data were then submitted to analysis of variance and the means were compared by the Tukey test (p ≤ 0.05). Statistical analyses were performed using the *easyanova* package (Arnhold, 2013) implemented in the R software (R Development Core Team, 2016).

### 2.4 Evaluation of the development of the plants in the field

After the nursery phase, the grafted and ungrafted plants, aged around 80 days and about 45 cm in height, were transplanted to the field of Embrapa Mandioca e Fruticultura. The experiment was carried out in a completely randomized block design, with 12 treatments distributed in three blocks (replicates), with plots of ten plants. The spacing used was 1.0 m between rows and 0.9 m between plants. The crop management was carried out according to cassava recommendations (Souza and Farias, 2006).

The male and female flowers and fruits were counted every 15 days starting 132 days after transplanting (September 2015), when the plants began to bloom. In order to verify changes in the grafted plants at 14 months after planting (harvest time), six agronomic traits were measured. Six of the twelve treatments that did not have the wild species FLA05-2 as scion or rootstock were evaluated: Self-0823, 0823/Formosa, 0823, Self-Formosa, Formosa/0823 and Formosa. Since FLA05-2 does not develop tuberous roots, the treatments derived from this accession were not evaluated.

The traits evaluated at harvest: total fresh root yield (FRY - measured in t ha^−^¹); aboveground yield (AGY - measured in t ha^-1^); harvest index (HI - ratio between FRY and total plant biomass in fresh weight); dry matter content in the roots (DMC - expressed as percentage and measured with the specific gravimetric method, according to Kawano et al., 1987); starch yield (STY - measured in t ha^-1^, considering starch content and FRY); and plant height (PH - measured in meters).

For all traits, the data were analyzed to test the assumptions for analysis of variance. For number of male and female flowers and number of fruits per plant, the assumption of homogeneity of residual variance was not met, requiring the transformation of the data using the Box-Cox method (Asar et al., 2017). After the analysis of variance and determination of the significance of the variation sources, the treatments were grouped by the Scott-Knott test (p ≤ 0.05). The statistical analyses for flowering and agronomic traits were performed using the easyanova package (Arnhold, 2013) implemented in the R program (R Development Core Team, 2016).

### 2.5 Weather conditions during the experiments

**Fig. 2:**
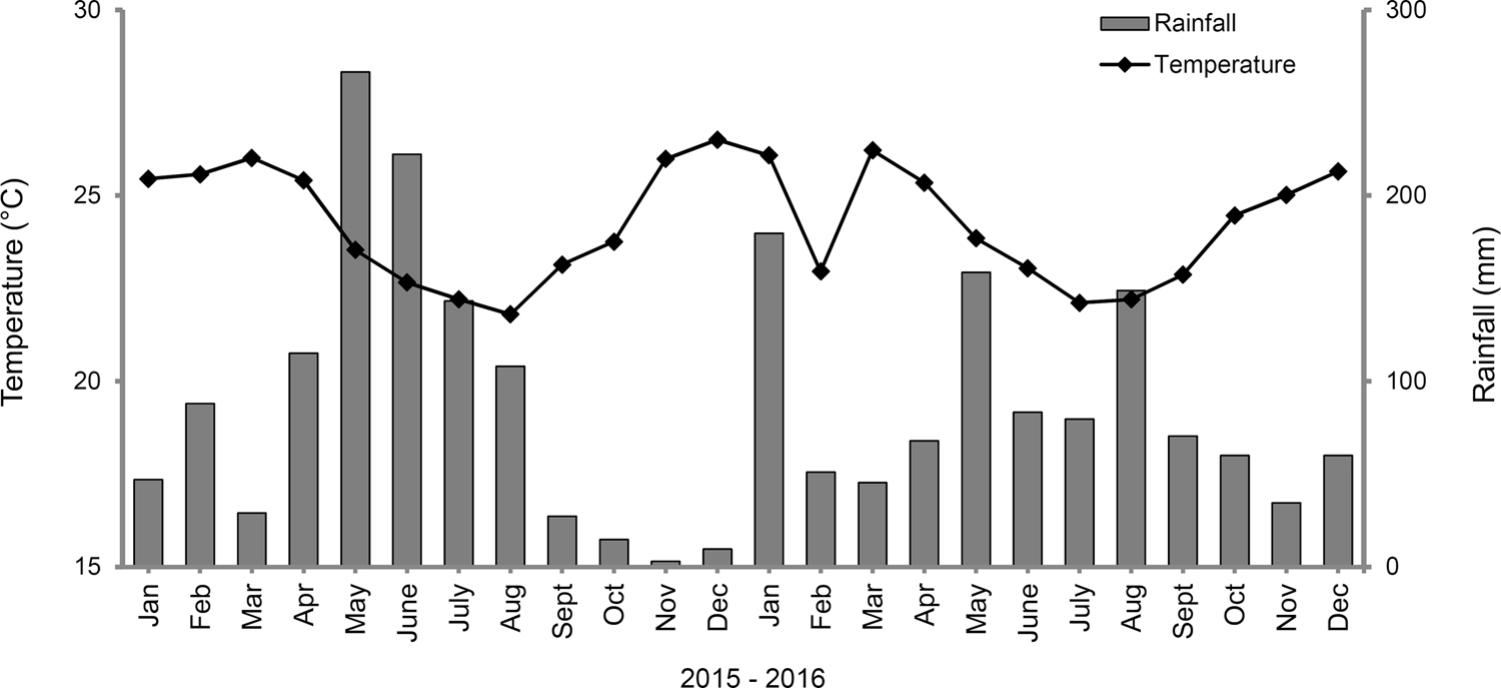
Average rainfall (mm) and air temperature (°C) in the field of Embrapa Mandioca e Fruticultura, Cruz das Almas, Bahia, during experimental period. Data collection period from flowering to harvest: September 2015 to May 2016.

The data on average rainfall and temperature of each month from January 2015 to December 2016 (flowering period) were obtained from the Embrapa Mandioca e Fruticultura meteorological station (Fig. 2). The longest day duration was recorded from October 2015 to February 2016 (ranging from 12.13 to 12.36 h of light), while the shortest day duration was recorded from April to July 2016 (ranging from 11.64 to 11.78 h of light).

## 3 Results

### 3.1 Viability of grafting

The total average grafting survival rate was approximately 70%, considering all treatments (Table 1). Therefore, the cleft grafting method can be used efficiently for the production of different scion-rootstock combinations. This combination had little influence on the survival rate, since there was no difference between most of the scion and rootstock combinations (Table 1). On average, the survival rate of the grafted genotypes ranged from 60% to 82%. Significant differences were observed only in the combinations with FLA as scion, in which the self-graft provided the highest survival rate (88%). On the other hand, the combination FLA/Formosa presented significantly lower survival rate than self-grafting (46%).

**Table 1:** Grafting survival rate of different combinations of graft and rootstock at 30 days after cleft grafting.

Rootstock	Scion		
	BRS Formosa	BGM0823	FLA05-02
BRS Formosa	77.2 Aa^a^	78.5 Aa	46.1 Bb
BGM0823	65.4 Aa	65.1 Aa	60.0 ABa
FLA05-02	67.0 Aa	82.2 Aa	88.3 Aa

a Means followed by the same letter, uppercase in the column and lowercase in the row, do not differ statistically from each other by the Tukey test at 5% probability.

The incompatibility presented by these grafted genotypes resulted in low plant development and may be related to physiological differences in stem diameter as well as to anatomical differences. Another hypothesis to explain the difference in the survival rate involving FLA05-02 refers to the fact that its propagation was by true seeds (sexual) and not by cutting (asexual), as done with BRS Formosa and BGM0823. Thus, greater variation among the FLA05-02 plants was expected because they were half-sib progenies. In other words, the great variation in the grafting survival may have been due to the lower uniformity of the seed-derived plants. In addition, FLA05-02 is another subspecies (*M. esculenta* ssp. *flabellifolia*), so there may have been genetic incompatibility in the grafting process. Moreover, *M. esculenta* ssp. *flabellifolia* may present wider genetic differences because of its phylogenetic distance from BRS Formosa and BGM0823. Indeed, according to Olsen (2004), *M. esculenta* ssp. *flabellifolia* presents shrub growth, less suberized root production and more elongated internodes, which is not common in *M. esculenta*.

### 3.2 Flowering analysis

Significant differences (p ≤ 0.01) among the twelve grafting treatments for all evaluated traits were found (Table 2). At least one treatment stood out from the others by having a greater number of male and female flowers and fruits. From the experimental point of view, the coefficient of variation (CV) was low for most of the traits associated with flowering (ranging from 5.4 to 14.32%), while the overall means for number of male flowers (NMF), number of female flowers (NFF) and number of fruits (NFT) were 97.82, 13.24 and 20.71, respectively.

**Table 2:** Summary of analysis of variance for number of male flowers (NMF), number of female flowers (NFF) and number of fruits (NFT) per plant for different combinations of grafts and rootstocks evaluated 14 months after planting.

Source of variation	DF^a^	Mean square		
		NMF	NFF	NFT
Blocks	2	1.4306	0.0104	0.0045
Treatments	11	105.5341**	0.8302**	0.0552**
Error	19	7.9712	0.0604	0.0058
Overall mean		97.82	13.24	20.71
CV (%)		14.32	10.25	5.41

a DF: degrees of freedom; ** significant at 1% probability by the F-test.

**Fig. 3:**
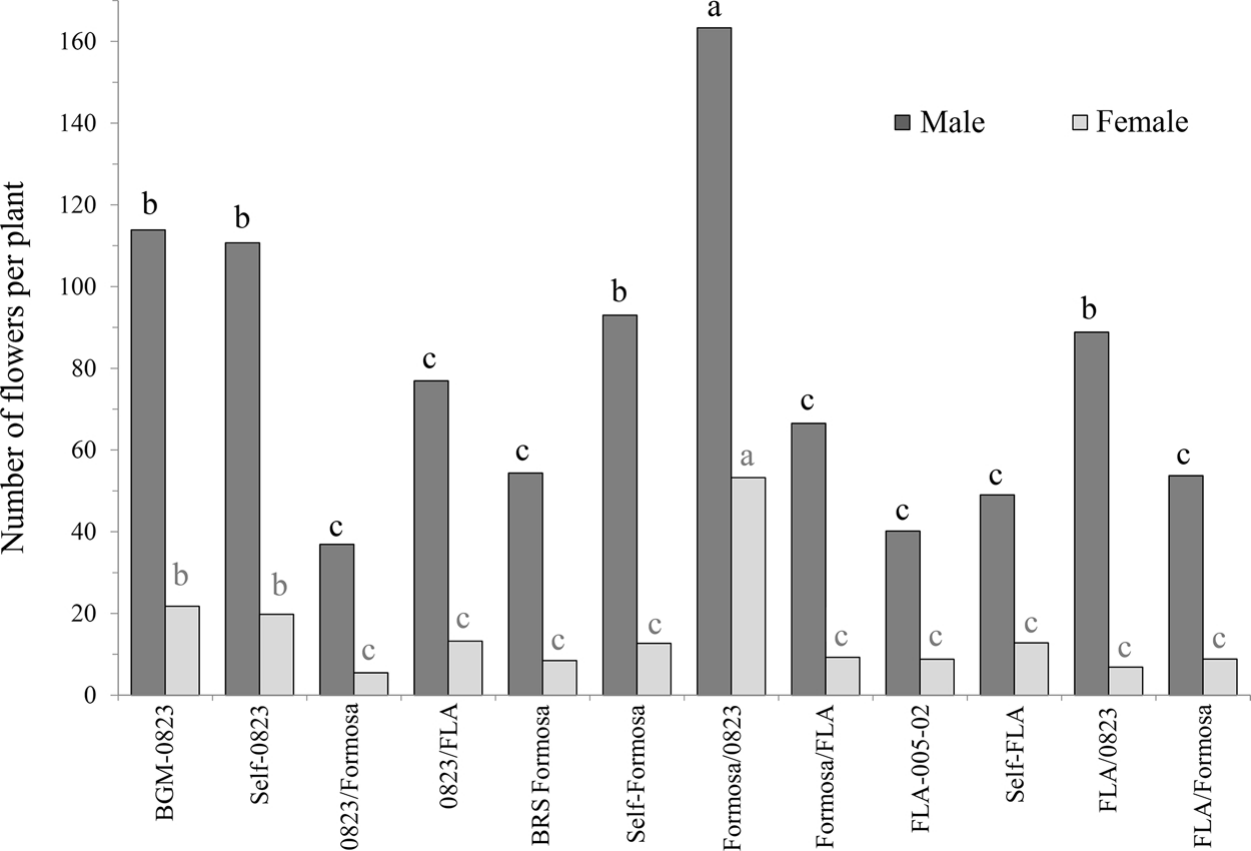
Average number of male and female flowers per plant in different combinations of grafting between genotypes with high (BGM0823 and FLA05-02) and low flowering rate (BRS Formosa) evaluated from September to December 2015 (five months after transplanting). Means followed by the same letters belong to the same group by the Scott-Knott test (p ≤ 0.05).

**Fig. 4:**
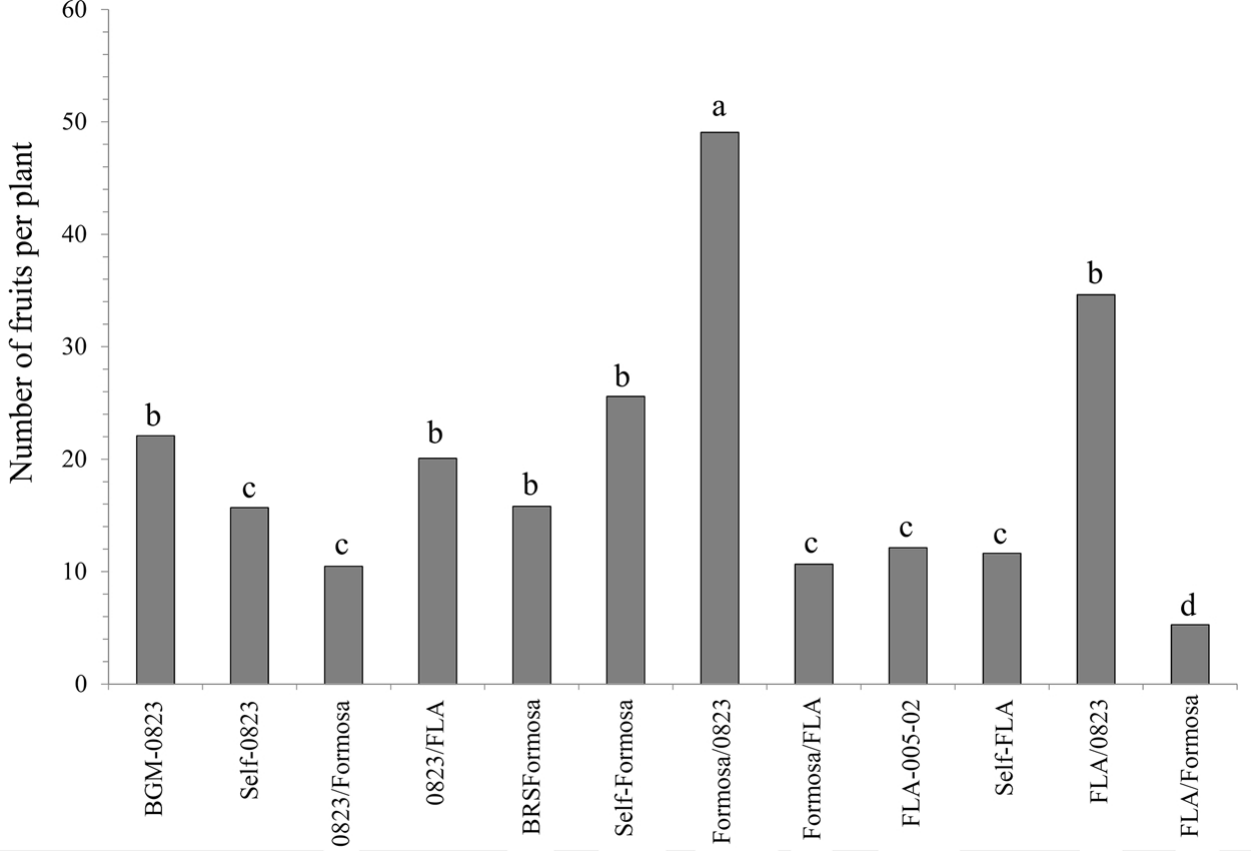
Average number of fruits per plant in different combinations of grafting between genotypes with high (BGM0823 and FLA05-02) and low flowering rate (BRS Formosa) evaluated from September to December 2015 (five months after transplanting). Means followed by the same letters belong to the same group by the Scott-Knott test (p ≤ 0.05).

In general, the average NMF, NFF and NFT values were higher in treatments where BGM0823 was used as rootstock (Self-0823, FLA/0823 and Formosa/0823), i.e., this genotype increased the number of male and female flowers and fruits of grafted plants (Fig. 3, Fig. 4). The graft of the genotype BRS Formosa on BGM0823 rootstock (Formosa/0823) showed higher production of flowers and fruits compared to the other treatments. Increases of 201% for NMF, 560% for NFF and 400% for NFT were recorded in relation to ungrafted treatments of BRS Formosa (Formosa) (Fig. 3, Fig. 4). Moreover, the performance of the FLA05-02 scion on the rootstock BGM0823 (FLA/0823) was also promising, possibly as a result of the floral stimulus, resulting in increases in NMF and NFT of 100%. The combination FLA/0823, therefore, doubled the production of male flowers in relation to the ungrafted treatment (FLA). However, for NFF this treatment was not beneficial due to the different flowering pattern between the two species.

Considering the production of male and female flowers and fruits among the ungrafted plants, 0823 was superior to the other genotypes. This result confirms previous observations regarding the profuse flowering habit and abundant fruit set of this genotype. FLA is also known as a species that flowers abundantly. However, in this study FLA produced fewer flowers than the other genotypes. A feasible explanation is that this genotype has a later flowering habit, that is, flowering gradually increases throughout the plant’s growth.

Regarding self-grafting, there was higher production of male flowers in Self-0823 and Self-Formosa treatments, compared with Self-FLA. In addition, for NFF the Self-0823 treatment was superior to Self-FLA and Self-Formosa. Finally, NFT in the Self-Formosa treatment was higher than the Self-0823 and Self-FLA treatments. Therefore, there was likely a higher rate of flower-fruit abortions in Self-0823. Apparently self-grafting improved fruit and flower production, possibly due to stress caused by the mechanical injury during the cleft grafting process, which interrupted the flow of water and nutrients. In this case, the increase in the production of flowers may be an adaptive response of the plant to the abiotic stress experienced.

### 3.3 Effects of grafting on agronomic traits of cassava

The agronomic traits of the grafted cassava plants were evaluated to verify the occurrence of changes in the performance of the plants after the grafting process. Significant differences were observed among the grafting treatments for above ground yield (AGY) and harvest index (HI) (Table 3). For the other traits, the treatments did not differ. Estimates of CV in relation to the dispersion of the data in terms of the mean value showed little variation between the traits, ranging from 7% (HI) to 23% (STY).

**Table 3:** Summary of analysis of variance for six agronomic traits of grafted cassava plants evaluated 14 months after planting.

Source of variation	DF^a^	Mean square					
		FRY^b^	AGY	STY	DMC	HI	PH
Blocks	2	2.70	1.09	0.41	0.25	0.35	0.31
Treatments	5	1.92^ns^	5.21*	0.72^ns^	0.06^ns^	1.24*	0.35^ns^
Error	10	1.19	1.13	0.62	0.36	0.24	0.11
Average		35.55	49.90	11.48	32.25	42.00	2.83
CV (%)		18.32	15.41	23.04	10.48	7.34	11.26

a DF: degrees of freedom.

b FRY: total fresh root yield (t ha^−1^); AGY: aboveground yield (t ha^−1^); STY (t ha^−1^): starch yield; DMC: dry matter content in the roots (%); HI: harvest index (%); PH: plant height (m); ^ns, *^: not significant and significant at 5% probability by the F-test, respectively.

Regarding AGY, two groups were formed, and the highest averages were observed in the treatments where BGM0823 was ungrafted (0823), self-grafted (Self-0823) and used as rootstock (Formosa/0823) (Table 4). The opposite was observed for treatments in which BRS Formosa was used as rootstock, demonstrating that the AGY is associated with the rootstock, and therefore has a strong genetic component. With respect to ungrafted treatments Formosa and 0823, this second accession produced 31 t ha^−1^ more aerial part than the first one.

**Table 4:** Average of two agronomic traits of grafted cassava plants evaluated 14 months after planting.

Treatments	Agronomic traits^a^	
	AGY	HI
Self-0823	31.81 a^b^	37.50 b
BGM0823	38.33 a	40.57 b
Formosa/0823	27.89 a	29.21 c
0823/Formosa	14.70 b	50.11 a
Self-Formosa	15.95 b	49.50 a
BRS Formosa	21.02 b	52.80 a

a AGY: aboveground yield (t ha^−1^); HI: harvest index (%).

b Means followed by the same lowercase letter in the column belong to the same group by the Scott-Knott test (p ≤ 0.05). Self-0823: self-grafted BGM0823; 0823/Formosa: BGM0823 (scion) x BRS Formosa (rootstock); BGM0823: BGM0823 (ungrafted); Self-Formosa: self-grafted BRS Formosa; Formosa/0823: BRS Formosa (scion) × BGM0823 (rootstock); BRS Formosa: BRS Formosa (ungrafted).

The productivity of BRS Formosa grafted on BGM0823 (Formosa/0823) was 50% higher than the reverse grafting (Table 4). The accession BGM0823 increased the AGY of BRS Formosa from 21.0 to 28.0 t ha^−1^ while in the reverse grafting process (0823/Formosa), this measure decreased from 38.0 to 14.0 t ha^−1^.

Similar results to AGY were observed for HI, in which two groups – (Sefl-0823, 0823 and Formosa/0823) and (0823/Formosa, Self-Formosa and Formosa) – presented similar within-group but different between-group averages (Table 4). For HI, the highest values were observed for Formosa (52.8%), 0823/Formosa (50.1%) and Self-Formosa (49%). HI is an important variable, since it provides the rate between total fresh roots produced in comparison with the total biomass of the plant (roots, stems and leaves). Since AGY is a major component of HI, these two traits had similar performance.

## 4 Discussion

### 4.1 Efficiency of cleft grafting in cassava

The survival rate obtained by cleft grafting ranged from 46% to 88%, depending on the scion-rootstock combination. Comparisons of the efficiency of this method are difficult because of the limited information about grafting of cassava. One of the few recent works on the subject used the splice grafting technique in adult plants (4 and 5 months) instead of seedlings (Ceballos et al., 2017). These authors evaluated six cassava genotypes with low flowering potential as scions and another genotype with high flowering potential as rootstock, and observed variation in the survival rate from 37% to 87%. According to them, the low survival of the scions can be explained by the stress caused by the grafting procedure as well as the malformation of the vascular tissue at the junction between the scion and rootstock, damaging the phloem and xylem flow. Therefore, the survival rate of 70% indicates that the technique is suitable for induction of flowering in cassava with a simple protocol.

Grafting of cassava has been described in the literature for objectives other than increased flowering. Grafting has been extensively used in diagnostic protocols for different cassava diseases. It has also been used on stems of adult plants to verify the compatibility of scions, whose results demonstrated 100% survival rate of the genotypes used as scions and the species *M. glaziovii* Muell as rootstock, and 30% success with the genotype as scion and *M. fortaleezensis* species as rootstock (Nassar et al., 2011). Therefore, the variation found in the survival rate showed that *M. fortaleezensis* presented greater incompatibility than *M. glaziovii* Muell. However, the difference between the survival rate was higher among the genotypes in this study, and the survival rates of the present study were different in magnitude than the findings of Nassar et al. (2011), obtained under other physiological conditions.

FLA05-02 presented some degree of incompatibility when grafted on BRS Formosa. Possibly there was low anatomical affinity of the conducting vessels, impairing the normal flow of photoassimilates and the lignification of tissues of the rootstock and scion. On the other hand, FLA/0823 presented a higher average survival rate compared to FLA/Formosa. According to Bomfim et al. (2011), there are anatomical differences in the diameter of the conducting vessels within the same cassava species. This might explain why two genotypes of *M. esculenta* had different results for survival rate using *M. esculenta* ssp. *flabellifolia* as scion. Future studies of the anatomy of conducting vessels between accessions of the same species will be crucial to indicate which combinations result in higher survival rates. In addition, the use of plants derived from different types of propagation [asexual (cutting) in the accessions of *M. esculenta* and sexual (true seeds) for accession FLA05-02] may have influenced the survival rate, considering that the seedlings are genetically different and may be less suitable than vegetatively propagated accessions. The incompatibility between scion and rootstock can be attributed to genetic variability in the plants grown from seeds. Since cassava is a highly heterozygous species, each seed is a unique genotype subject to specific interactions with other environmental factors.

### 4.2 Efficiency of grafting as a method for producing flowers and fruits

Generally, grafting has several purposes in many plant species, such as enhanced tolerance to soil diseases and abiotic stress, promotion of plant vigor, increased yields and better fruit quality. It can also be used in physiological studies, such as investigation of flower induction and early flowering (Lee et al., 2010). Grafting methods have been investigated to induce or increase flowering rate in various crops, such as potato (Lardizabal and Thompson, 1990) and sweet potato (Mubayiwa et al., 2016). Grafting has also been efficiently exploited to accelerate flowering in gourds (Lin et al., 2007), tomato and tobacco (Lifschitz et al., 2006).

An unexpected result was observed with FLA05-02, whose flower and fruit increments in the grafting treatment were not as high as those with BGM0823. The fact that FLA05-02 is a wild species and has a late flowering habit may explain these results. The variation in the response of the treatments showed that despite being a complex trait, flowering in cassava can be induced by grafting, especially within species.

Because of the scarcity of reports of the efficiency of grafting for the induction of flowering in cassava, the results of the present work are an important contribution to knowledge about more efficient strategies to expand the possibilities of crosses between cassava genotypes. Ceballos et al. (2017) performed splice grafting in adult plants using stems from a non-flowering genotype as scion and plants with profuse and early branching as rootstock and observed no improvement in flowering. However, when stems from grafted plants were planted, the authors verified that at 174 days after planting, the plants derived from the grafted aerial part showed an increase in the production of flowers, fruits and seeds in relation to ungrafted plant cuttings in one genotype. As in the present study, therefore, these authors found genetic variation in the response to grafting. They ascribed this to the memory effect on the grafted stems. Therefore, the planting of the grafted stems obtained in the present work should be studied further to determine if the memory effect can bring more gains in the induction of flowering of low-flowering genotypes like BRS Formosa.

Despite the promising results in the induction of flowering using grafting under field conditions, Ceballos et al. (2017) reported some inefficiency of the method. In addition, under glasshouse conditions, Bull et al. (2017) evaluated adult plants of two transgenic cassava genotypes with profuse flowering as rootstock and the non-transgenic one with poor flowering as scion. The results showed that none of the grafted plants presented significant increases in the number of male and female flowers and fruits, regardless of the scion-rootstock combination used. According to Bull et al. (2017), the rootstock was not able to generate sufficient quantities of the flowering stimulus, that is normally loaded in the phloem. These reports make important contributions to the generalization of this research, since aspects such as genotype, environment (growth and analysis in the field or greenhouse) and even genetic (transgenic vs. conventional) will certainly need to be analyzed in more detail in future research.

According to Giakountis and Coupland (2008), the flowering process is driven by stimuli where a set of substances together with the FT form a signal that is carried by the phloem to the apical shoot. With this, an expression reaction is initiated that triggers the molecular apparatus and stimulates floral initiation in the apical meristem. Also, according to those authors, grafting can be used for transmission of the flowering signal between rootstock and scion. In this sense, we believe that the flowering stimulus of BGM0823 was transmitted during the process of establishing and joining the scion, since the basal leaves of the rootstock were conserved for approximately 15 days until the success of the scion, being removed from the plants after this time.

For induction of flowering in tomato, cleft grafting was performed aiming to transmit the flowering stimulus and the photoassimilates produced by the leaves through the phloem to the apical meristem of a host or rootstock that showed low flowering rate (Lifschitz et al., 2006). According to the authors, the flow of photoassimilates via the phloem from the basal leaves of the rootstock apparently passes into the scion, also carrying the flowering stimulus between the leaves, so that it is accumulated in the apical meristem of the grafted plants as a “flowering mark”. Thus, this mark probably remains in an inactive form that is activated after exposure of the stimulus to ideal conditions of light and temperature for flowering, that is, a “memory effect” condition can be created. The memory effect according to Bruce et al. (2007) is considered an epigenetic mark that can be left in a transcriptionally silent state in which the bud region of the apical meristem is in a “permissive” state. According to the same authors, at the appropriate moment of flowering this condition allows a response, and expression of the characteristic occurs, initiating flowering.

In addition to the memory effect, another plausible and complementary explanation for the induction of flowering in cassava by grafting is related to the role of roots, considering their integration in the regulation network of the systemic signaling of flowering through the action of global genes involved in the induction of flowering (Bouché et al., 2016). Thus, probably the floral stimulus regulated by BGM0823 as a rootstock on FLA05-02 and the BRS Formosa genotype operated in two strands, the first one associated with the movement of photoassimilates by the leaf phloem, in which the FT may be one; and the second where the key regulators are probably expressed in the roots. However, further studies in this respect still need to be carried out.

As shown, grafting can be used to induce flowering in cassava plants. However, the time it takes for plants to emit flowers after grafting can be a determining factor in hybridization processes. According to Ceballos et al. (2017), the total time for plants to start flowering was 389 days after grafting and planting the cuttings in the field. However, in the present work, grafted plants were transplanted directly to the field, and the beginning of flowering occurred about 210 days after grafting. In this sense, the precocity of flower production using the grafting in seedlings was approximately 179 days when compared to the grafting of adult plants used by Ceballos et al. (2017). Thus, efficiency in cassava grafting conducted in the nursery could provide crop breeders with a gain in time to establish plant crosses in breeding programs. Another advantage would be the greater safety of grafting conducted in the nursery than in the field, especially regarding problems like loss of scions by rupture of the grafted site due to the action of the wind or traffic of people, besides the greater control over pests and diseases.

### 4.3 Effect of grafting on agronomic traits

Since the 1970s, several researchers have used grafting methods to increase root production of cassava. In these studies, genotypes with high photosynthetic potential were used as scions and genotypes with high fresh root yield as rootstock (Ahit et al., 1981; Dahniya et al., 1982; Ramanujam and Ghosh, 1990). The results showed that grafting in adult plants did not influence the production of roots, starch and dry matter content, similar to the results found in the present study.

The cleft grafting promoted significant changes in AGY, as observed in the Formosa/0823 combination, with an increase of about 25% in relation to ungrafted BRS Formosa. On the other hand, reverse treatment (0823/Formosa) reduced AGY by approximately 50% compared to ungrafted BGM0823. The root system of the rootstock can be a determining factor to increase the production of the aerial part, since roots with better development capture larger amounts of water and nutrients. For melon (*Cucumis melo* L.), Salehi-Mohammadi et al. (2009) observed that the rootstock is mainly responsible for the expression of the aerial part and fruits of the grafted plants.

The increase of the AGY in the scions of BRS Formosa did not result in an increase in the FRY of the rootstock BGM0823, indicating that the development of shoot and roots in grafted plants did not present any positive relation. The increase in the AGY provided by BGM0823 rootstock can be explained by the specific genetic traits of this accession, which can modify physiological and biochemical patterns of the BRS Formosa scions in order to increase biomass gains. On the other hand, on average the AGY in self-treatments in both BGM0823 and BRS Formosa did not increase in relation of ungrafted plants.

Even though grafting increased or reduced AGY in some treatments, this pattern was not observed for FRY. Therefore, on average FRY did not show significant differences between grafted and ungrafted treatments, indicating that the root production potential of each accession is mostly defined by the genetic background. In other words, the sink strength is genetically fixed in the rootstock. On the other hand, HI was the agronomic trait that presented the most significant difference between treatments. Considering that the aerial part presents a direct relation with the rootstock, being influenced positively or negatively depending on the scion-rootstock combination, it can be inferred that the observed differences between the treatments for HI were more influenced by AGY than by FRY.

## 5 General considerations on the use of grafting for flowering induction in cassava

The use of grafted cassava plants in the early stages of development is a viable strategy due to the high survival rate, possibly because there is greater ease and speed in the joining between the vascular tissue of the scion and rootstock. The use of grafting as a tool to trigger endogenous reactions that induce dynamic changes in the scion response allowed inducing a higher flowering rate in cassava, which will certainly bring positive impacts from increasing the use of parent plants in the genetic breeding of the crop.

Increasing flowering represents a major gain in breeding programs since it facilitates routine cross-breeding operations in nurseries, reducing operating costs and accelerating the production of segregated populations, as well as facilitating the development of inbred parents and optimizing the process of genomic selection, which requires rapid generation of progenies for the various selection cycles (Ceballos et al., 2017). In addition, the induction of flowering in progenitors that do not normally produce flowers or produce them too late in the crop cycle could provide significant gains with the exchange of favorable alleles at an earlier and more efficient level. Finally, this study was carried out to investigate the functional and practical significance of the floral induction process through grafting. Our finding is that grafting in cassava, as reported here, is not a difficult process, because it requires only the same grafting care traditionally applied to fruit tree species, with low implementation cost, since it can be done in the nursery phase without the need for sophisticated structures. Above all, it is efficient enough to induce flowering in cassava, depending on the choice of scions. On the other hand, many molecular tools have been used to elucidate the mechanisms and genes that control flowering in cassava (Adeyemo et al., 2017; Bull et al., 2017). In the future it will be necessary to integrate efforts to investigate the metabolic pathways and genes that are involved in the flowering process, and how genetic effects that are still under study in cassava (epigenetic) might act in the control of this trait in cassava.

In conclusion, the production of grafted seedlings of cassava should be carried out in plants up to 30 days after rooting, so that a further 65% in the survival rate can be reached. At that age, the transfer of the flowering stimulus is quite efficient, resulting in the early induction of flowering, increasing flower and fruit production, as observed for the treatments in which BGM0823 was used as rootstock. Thus, grafting can be classified as a new tool to obtain improved genotypes in cassava and its use can accelerate the flowering process in a practical, fast inexpensive way. In this sense, it is important to highlight that in work of Ceballos et al. (2017), two branches of the rootstock were left untouched so that they would contribute a “continuous and strong” signal to the grafted part. In this study, on the other hand, two leaves were left in the rootstock for a brief period of time. Comparison of the results of the two contrasting approaches clearly shows there is no need for a “continuous and strong” signal for the induction of flowering.

## Conflicts of interest

The authors declare no financial or other competing conflicts of interest.
